# Hepatocellular carcinoma stem cells: the current state of small molecule-based inhibitors

**DOI:** 10.1038/s41419-025-07983-5

**Published:** 2025-09-01

**Authors:** Sara P. Neves, Larissa M. Bomfim, Daniel P. Bezerra

**Affiliations:** Gonçalo Moniz Institute, Oswaldo Cruz Foundation (IGM-FIOCRUZ/BA), Salvador, Bahia Brazil

**Keywords:** Pharmacology, Cancer stem cells

## Abstract

Hepatocellular carcinoma (HCC) is the most common type of liver cancer, accounting for over 90% of all cases. Patients with advanced-stage HCC are referred to systemic treatment. Although some advances in HCC therapy have been made in recent years, the prognosis for patients remains poor due to drug resistance, tumor relapse, and metastasis, implying that overall survival remains a challenge. Many studies have shown that tumor-initiating stem cells, also known as cancer stem cells (CSCs), play essential roles in tumorigenesis, metastasis, and treatment resistance in HCC and that future cancer treatments could be significantly improved by targeting this cell population subset. Different markers of CSCs from HCC have been identified, and intracellular signaling pathways and extracellular factors have been reported as targets capable of removing this cell subpopulation, highlighting the possibility of developing targeted drugs to eradicate HCC CSCs. In this review, we highlight emerging small compounds that target HCC CSCs to provide new insights and guide future research. Drugs in the preclinical and clinical trial development stages were selected and discussed.

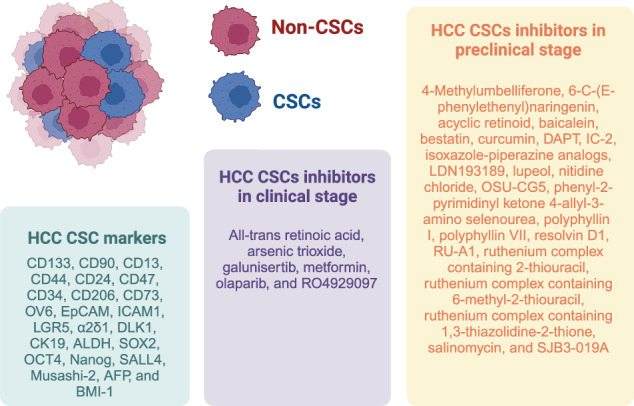

## Introduction

Liver cancer is a major global health problem, with 865,269 new cases and 757,948 deaths occurring in 2022 worldwide, making it the third most common cause of cancer-related death and the sixth most common type of cancer [1]. According to the United States of America surveillance, epidemiology, and end results (SEER) database, the 5-year relative survival rates for people diagnosed with liver/intrahepatic bile duct cancer between 2014 and 2020 were 37% for localized cancers, 13% for regional cancers, and 3% for distant cancers, resulting in 22% for all stages combined [2].

Liver cancer includes hepatocellular carcinoma, cholangiocarcinoma, hepatoblastoma, and combined hepatocellular carcinoma and cholangiocarcinoma [3]. Hepatocellular carcinoma (HCC) is the most common type of liver cancer, accounting for ~90% of cases. Infection with hepatitis B and C viruses is the primary risk factor for the development of HCC. However, nonalcoholic steatohepatitis, often linked to metabolic syndrome or diabetes mellitus, is increasingly becoming a more common risk factor [4–7].

The complexity and diversity of HCC can be attributed to the distinct genetic and molecular profiles present in the disease, ranging from early to advanced stages. Mutations in the *TERT*, *TP53*, and *CTNNB1* genes are frequently found in HCC [8]. On the other hand, several molecular pathways, including the Wnt/β-catenin, oxidative stress, epigenetic regulation, PI3K/Akt/mTOR, and MAPK pathways, are responsible for hepatocarcinogenesis [9, 10].

Patients with early-stage HCC should be assessed for curative treatment options such as liver transplantation, hepatectomy, or ablation [11]. For patients with intermediate-stage HCC, transarterial radioembolization or chemoembolization are the most common therapeutic modalities. Patients with advanced-stage HCC are referred for systemic therapy [12]. In addition to conventional chemotherapeutics (e.g., doxorubicin, cisplatin, oxaliplatin, and 5-fluorouracil), targeted therapy drugs include kinase inhibitors (e.g., sorafenib, regorafenib, lenvatinib, and cabozantinib), immune checkpoint inhibitors (e.g., pembrolizumab, atezolizumab, and nivolumab), and angiogenesis inhibitors (e.g., ramucirumab and bevacizumab) and are currently used for systemic therapy in patients with HCC. In patients with advanced HCC, only targeted drugs are used as first- and second-line treatments [13–15].

Despite significant advances in the treatment of HCC, the prognosis for patients remains poor due to drug resistance, tumor recurrence and metastasis, meaning that overall survival remains unsatisfactory. Several studies have revealed the presence of tumor-initiating stem cells, also known as cancer stem cells (CSCs), which are believed to play critical roles in tumorigenesis, metastasis, and drug resistance in HCC [16–18]. In this review, we discuss emerging small molecules that target HCC CSCs to provide a new perspective and drive future research.

## Cancer stem cells from hepatocellular carcinoma

CSCs are a unique subset of cells within a tumor that exhibit characteristics like those of normal stem cells and present three main characteristics: tumor-initiating ability, self-renewal, and differentiation [19–21]. They were first identified in 1994 in leukemic cells with the CD34+CD38− phenotype [22] and have since been identified in different types of cancer.

CSCs are among the main causes of tumor heterogeneity and are also responsible for an aggressive phenotype, recurrence, therapeutic resistance and metastasis due to the phenotypic plasticity of these cells within the tumor [23–25]. CSCs also possess metabolic plasticity and the ability to constantly change their metabolic state between glycolysis and oxidative metabolism to adapt to nutrient deficiency and therapeutic stress [21, 26–28]. CSC resistance to conventional treatments, such as chemotherapy and radiation therapy, is attributed to various mechanisms, including enhanced DNA repair, drug efflux pumps, and the activation of survival signaling pathways. Additionally, CSCs can enter a dormant state, allowing them to evade the effects of therapy and later reactivate to regenerate the tumor [21, 29].

Some biomarkers have been identified to isolate and characterize CSCs in HCC (Table 1). Hepatoblasts and liver progenitor cells also express most of these markers, whereas mature adult hepatocytes do not. Many studies have shown that increased CD133 expression in HCC is a risk factor for overall survival and recurrence-free survival [30–32]. Furthermore, Wang et al. [33] reported that a phase 2, open-label, single-arm clinical trial of CD133-targeted chimeric antigen receptor T (CAR-T) cell treatment was effective and safe for patients with advanced HCC. Huang et al. [34] demonstrated that an anti-CD3/anti-CD133 bispecific antibody eliminated CD133^high^ HCC CSCs in vitro and in vivo, reducing tumor growth. Similar results were observed with oncolytic viruses that target CD133 [35] and with dendritic cell-based vaccines [36].

**Table 1:** HCC CSC markers.

HCC CSC markers	Full name or alternative names	Function	References
***Cell surface markers***			
CD133	Prominin-1 PROM1	Membrane glycoprotein associated with the maintenance of cell membrane integrity	[251]
CD90	Thy-1	Cell surface glycoprotein involved in cell adhesion and signaling	[37]
CD13	alanyl aminopeptidase, membrane ANPEP	Aminopeptidase N, a cell surface enzyme involved in regulating the activity of bioactive peptides	[67]
CD44	Homing Cell Adhesion Molecule Phagocytic Glycoprotein-1	Cell surface glycoprotein involved in cell‒cell interactions, adhesion and migration	[37]
CD24	Heat Stable Antigen	Cell surface glycoprotein involved in cell adhesion and signaling	[252]
CD47	CD47 molecule	Cell surface glycoprotein related to inhibition of phagocytosis	[253]
CD34	CD34 molecule	Cell surface glycoprotein associated with cell adhesion and signaling	[254]
CD206	mannose receptor C-type 1 MRC1	Mannose receptor, a cell surface lectin involved in carbohydrate endocytosis	[255]
CD73	5’-nucleotidase ecto NT5E	Ectoenzyme that converts AMP to adenosine, modulating cell signaling	[256]
OV6	Ov6 protein Oval Cell Marker	Ovarian stem cell-specific cell surface antigen	[257]
EpCAM	Epithelial Cell Adhesion Molecule CD326	Cell surface glycoprotein related to cell adhesion and signaling	[38]
ICAM1	Intercellular Adhesion Molecule 1 CD54	Cell adhesion molecule involved in cell‒cell interaction and immune response	[258]
LGR5	Leucine Rich Repeat Containing G Protein-Coupled Receptor 5 G-Protein Coupled Receptor 49 G-Protein Coupled Receptor 67	G protein-linked G protein receptor involved in Wnt signaling	[259]
Calcium channel α2δ1	Calcium voltage-gated channel auxiliary subunit alpha2delta 1 CACNA2D1	Auxiliary subunit of calcium channels, involved in the modulation of cell signaling	[260]
DLK1	Delta-like 1 noncanonical Notch ligand 1	Membrane protein involved in Notch signaling	[261]
***Intracellular markers***			
CK19	cytokeratin 19 Keratin 19	Cytoskeletal protein associated with cell differentiation	[181]
ALDH	Aldehyde Dehydrogenase	Enzyme that oxidizes aldehydes and retinol, involved in stem cell differentiation	[262]
SOX2	SRY-Box Transcription Factor 2	Transcription factor involved in maintaining pluripotency and self-renewal of stem cells	[42]
OCT4	Octamer-Binding Transcription Factor-4 POU Domain, Class 5, Transcription Factor 1	Transcription factor involved in maintaining pluripotency and self-renewal of stem cells	[42]
NANOG	Nanog Homeobox	Transcription factor involved in maintaining pluripotency and self-renewal of stem cells	[43]
SALL4	Sal-Like Protein 4	Transcription factor involved in maintaining pluripotency and self-renewal of stem cells	[44]
Musashi-2	RNA-Binding Protein Musashi Homolog 1/2	Protein associated with the maintenance of pluripotency and self-renewal of stem cells	[45]
AFP	Alpha Fetoprotein	Protein associated with cell differentiation	[263]
BMI-1	B Lymphoma Mo-Mlv Insertion Region 1 Homolog Polycomb Group RING Finger Protein 4 RING Finger Protein 51	Transcriptional repressor involved in maintaining pluripotency and self-renewal of stem cells	[46]

Similarly, Yang et al. [37] reported that CD90+ cells from HCC cell lines, but not CD90− cells, demonstrated tumorigenicity. CD90+CD44+ cells exhibit more aggressive behavior than CD90+CD44− cells do, forming metastatic lesions in the lungs of immunodeficient animals. The inhibition of CD44 activity by the administration of an anti-CD44 antibody also inhibited the development of local and metastatic tumor nodules by CD90+ cells. Similarly, Yamashita et al. [38] demonstrated that EpCAM+ HCC cells also have similar characteristics to CSCs, including a high capacity to generate HCC in immunodeficient animals. High protein expression levels of HCC CSC markers, such as CK19, CD133, and CD44, are strongly linked to tumor angiogenesis and a poor prognosis for patients with HCC [39]. Genetic signatures linked to CK19 have also been shown to predict the prognosis of HCC patients undergoing liver transplantation [40].

Yamashita et al. [41] studied HCC tissues and cell lines and reported that EpCAM+ cells had poor morphology and elevated serum alpha fetoprotein (AFP), whereas CD90+ cells had a high metastatic rate, suggesting two distinct hepatic CSCs, in which EpCAM+ cells exhibit epithelial characteristics, whereas CD90+ cells exhibit mesenchymal characteristics. In serial xenografts of primary HCCs from immunodeficient mice, EpCAM+ cells proliferate rapidly, whereas CD90+ cells have a strong metastatic capacity in the lungs.

**Fig. 1:**
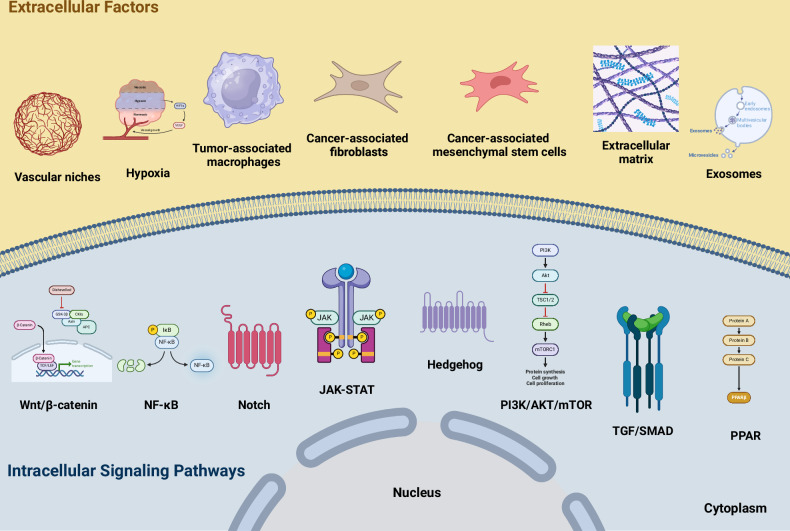
Illustration of CSC regulators. CSCs are regulated by intracellular signaling pathways and extracellular factors, such as vascular diseases, hypoxia, tumor-associated macrophages, fibroblasts, cancer-associated mesenchymal cells, the extracellular matrix, and exosomes. Created with BioRender.com.

At the molecular level, CSCs are regulated by pluripotent transcription factors such as OCT4, SOX2, NANOG, KLF4, and MYC [42–47]. In addition, several intracellular signaling pathways, such as the Wnt/β-catenin, NF-κB, Notch, Hedgehog, JAK/STAT, PI3K/Akt/mTOR, TGF/SMAD, and PPAR pathways, are responsible for the regulation of these cells [20, 48–50]. Moreover, extracellular factors, including vascular niches, hypoxia, tumor-associated macrophages, cancer-associated fibroblasts, cancer-associated mesenchymal stem cells, the extracellular matrix, and exosomes, are also important regulators of CSCs [19–21]. Figure 1 illustrates many CSC regulators. Therefore, several molecules have been identified as potential drugs to eradicate HCC CSCs.

## Small molecules targeting HCC CSCs

A total of 30 small molecules targeting HCC CSCs were selected for discussion, including preclinical and clinical data. Table 2 summarizes all the drugs discussed.

**Table 2:** Small molecules targeting HCC CSCs.

Drug	Molecular target	Main findings	References
4-Methylumbelliferone	Hyaluronan	Decreased CSC markers and prolonged survival in an orthotopic HCC model	[51]
6-C-(E-phenylethenyl)naringenin	Wnt/β-catenin pathway	Suppressed the stemness of HCC cells	[58]
Acyclic retinoid	MYCN	Reduced the number of EpCAM+ cells	[59]
All-trans retinoic acid (also known as tretinoin)	Akt pathway	Induced hepatic CSC differentiation	[138]
Arsenic trioxide	JAK1/STAT3, NF-κB and GLI1 pathways	Inhibition of the CSC properties in the multidrug-resistant HCC cell line MDR Bel-7402	[157, 159, 160]
Baicalein	Autophagy	Chemosensitized HCC CSCs	[62]
Bestatin (also known as ubenimex)	CD13	Chemosensitized HCC CSCs	[66]
Curcumin	NF-κB and PI3K/Akt/mTOR pathways	Suppressed HCC CSCs	[75, 76]
DAPT	Notch and Wnt pathways	Reduced EpCAM+ and EpCAM- Huh7 cell and prolonged survival in an in vivo HCC model	[81, 82]
Galunisertib (also known as LY2157299)	TGF-β pathway	CK19+ HCC cells suppressed in vitro and in vivo	[180, 181]
IC-2	Wnt pathway	Reduction of CSC markers and sphere-forming capacity as well as reduction of tumor growth in mice xenografted with CD44+ HuH7 cells	[90]
Isoxazole-piperazine analogs	–	Reduced stem cell markers and ability of HCC cells to form spheres	[93]
LDN193189	BMP pathway	Reduced self-renewal and tumorigenicity of hepatic CSC	[94]
Lupeol	PTEN/Akt/ABCG2 pathway	Suppressed liver CSC spheroids derived from HCC cell lines and clinical specimens	[99]
Metformin	Oxidative stress	Decreased CSCs from HCC biopsies	[193]
Nitidine chloride	STAT3 pathway	Decrease in EpCAM+/CD133+ HCC cells and inhibition of sphere formation	[104]
Olaparib	PARP	Suppressed pluripotent transcription factor in HCC tumors	[231]
OSU-CG5	mTOR pathway	Decreased the CD90 population in primary liver tumor samples and cell lines	[108]
Phenyl-2-pyrimidinyl ketone 4-allyl-3-amino selenourea	c-MYC	Sensitized tumors to sorafenib and suppressed HCC CSCs	[109]
Polyphyllin I	Akt/GSK-3β/β-catenin pathway	Inhibited HCC CSCs and reduced tumor growth in HCC cells in a xenograft model	[110]
Polyphyllin VII	STAT3 pathway	Inhibited migration, tumor spheroid formation and stem cell markers in HCC cells	[111]
Resolvin D1	CAFs	Prevented CAF-induced hepatic CSCs and the EMT of HCC cells	[113]
RO4929097 (also known as RG-4733)	Notch pathway	Reduced the expression of the hepatic progenitor cell markers	[240]
RU-A1	BMI1 pathway	Reduced the hepatic CSC population	[114]
Ruthenium complex containing 2-thiouracil	NF-κB and Akt/mTOR pathways	Reduction of clonogenic potential, expression of CD133+ and CD44^high^ cells, development of tumor spheroids and cell motility in HepG2 cells	[115]
Ruthenium complex containing 6-methyl-2-thiouracil	Akt/mTOR pathway	Reduction of clonogenic potential, expression of CD133+ and CD44^high^ cells, development of tumor spheroids and cell motility in HepG2 cells	[115]
Ruthenium complex containing 1,3-thiazolidine-2-thione	Akt/mTOR pathway	Inhibited clonogenic potential, CD133+ and CD44^high^ cells, tumor spheroid growth and cell motility in HepG2 cells	[116]
Salinomycin	FAK-ERK1/2 and Wnt/β-catenin pathways	Suppressed the ability of hepatic CSCs both in vitro and in vivo	[119, 120]
SJB3-019A	USP1	Decreased the sphere-forming ability and the cancer stemness of HCC cells	[136]

### Small molecules in the preclinical development stage

Most small molecules that target HCC CSCs have been found only in preclinical studies. These included 4-methylumbelliferone, 6-C-(E-phenylethenyl)naringenin, acyclic retinoid, baicalein, bestatin, curcumin, DAPT, IC-2, isoxazole-piperazine analogs, LDN193189, lupeol, nitidine chloride, OSU-CG5, phenyl-2-pyrimidinyl ketone 4-allyl-3-amino selenourea, polyphyllin I, polyphyllin VII, resolvin D1, RU-A1, ruthenium complex containing 2-thiouracil, ruthenium complex containing 6-methyl-2-thiouracil, ruthenium complex containing 1,3-thiazolidine-2-thione, salinomycin, and SJB3-019A. Although they have demonstrated promising ability to eradicate CSCs from HCC specimens and/or cultured cell lines, as well as in animal models, clinical trials using these molecules in HCC patients are needed to understand their true potential in HCC treatment.

#### 4-Methylumbelliferone

4-Methylumbelliferone is a hyaluronan synthesis inhibitor. Rodríguez et al. [51] reported that combining this molecule with an adenovirus carrying the interleukin-12 gene decreased the transcription of the CSC markers CD133, CD90, CD47, EpCAM, CD44, and CD13 in an orthotopic HCC model. Treatment with 4-methylumbelliferone also prolonged survival. This combination improved the response of specific cytotoxic T cells, and treatment with 4-methylumbelliferone decreased the number of CD47+ cells on HCC cells and caused phagocytosis by antigen-presenting cells. Additionally, Rodríguez et al. [52] reported that 4-methylumbelliferone treatment caused hepatic macrophages to adopt an M1-like phenotype. Interestingly, conditioned medium from these 4-methylumbelliferone-treated macrophages reduced tumor aggressiveness and downregulated the expression of many CSC markers in HCC Hepa129 cells. These cells delay tumor progression in immunocompetent mice but grow in immunodeficient mice.

4-Methylumbelliferone also prevents the expression of CSC markers during hepatocarcinogenesis [53]. CSCs from melanoma [54], breast [54], lung [55], oral squamous cell [56], and ovarian [57] cancers have also been reported to be affected by 4-methylumbelliferone, along with tolerable systemic toxicity.

#### 6-C-(E-phenylethenyl)naringenin

6-C-(E-phenylethenyl)naringenin, a seminatural derivative of naringenin (a type of flavonoid), suppresses the stemness of HCC cells by targeting the Wnt/β-catenin signaling pathway [58]. 6-C-(E-phenylethenyl) naringenin decreases viability and inhibits the migration, invasion, sphere formation, and epithelial‒mesenchymal transition (EMT) of Huh7 and Hep-3B HCC cells. Similar results were detected in NANOG+ cells sorted from cultured HCC cells. A reduction in HIF-1 activity and the expression of stemness-associated transcription factors were also detected. At the molecular level, the action of the molecule is mediated by downregulation of phospho-Akt, phospho-ERK, and phospho-GSK3β (Ser9), upregulation of GSK3β, induction of β-catenin degradation, and inhibition of its nuclear translocation. In addition, 6-C-(E-phenylethenyl)naringenin inhibited HCC tumor growth and lung metastasis in a mouse model of Huh7 cells without apparent systemic toxicity and increased the sensitivity of HCC cells to 5-fluorouracil, cisplatin, and sorafenib [58].

#### Acyclic retinoid

Acyclic retinoid is a retinoid derivative with cytotoxic properties. Qin et al. [59] reported that acyclic retinoid reduced the expression of MYCN, a member of the MYC family of basic helix-loop-helix-zipper transcription factors, in HCC cell cultures, animal models, and liver biopsies from HCC patients. Acyclic retinoid therapy inhibited cell growth and colony formation while inducing apoptosis in HCC cells. In HCC cells with elevated MYCN expression, acyclic retinoid reduces the number of EpCAM+ cells.

Qin et al. [60] demonstrated that acyclic retinoid binds directly to transglutaminase 2, promoting oligomer formation and inhibiting its cytoplasmic activity in HCC cells, resulting in cell death in a subpopulation of EpCAM+ hepatic CSCs. In normal cells, Guan et al. [61] reported that acyclic retinoid regulates retinoic acid receptors, which reduces the clonal proliferation of normal hepatic stem cells and promotes immature cell differentiation.

#### Baicalein

Baicalein is a flavonoid compound derived from the roots of *Scutellaria baicalensis*, a traditional Chinese plant. Interestingly, baicalein chemosensitized HCC CSCs by inhibiting autophagy via competitive inhibition of the guanosine triphosphatase SAR1B. Baicalein enhanced cell death induced by mTORC1 inhibition in HCC CSCs and Huh7 spheroid formation, as well as in a patient-derived xenograft (PDX) model of HCC, while maintaining a favorable safety profile [62].

Baicalein has also been reported to inhibit pancreatic CSCs via suppression of the hedgehog pathway [63] and T-cell lymphoma CSCs by reducing the thioredoxin system [64]. Colorectal CSCs are also inhibited by baicalein [65].

#### Bestatin

Bestatin, also known as ubenimex, is a metalloprotease inhibitor that is selective for aminopeptidases, including aminopeptidase N (CD13). Yamashita et al. [66] investigated the effects of bestatin combined with conventional chemotherapies, such as 5-fluorouracil, cisplatin, doxorubicin, and sorafenib, in the human HCC cell lines HuH7 and PLC/PRF/5. Interestingly, CD13 expression increased after exposure to each chemotherapeutic drug alone but decreased when combined with bestatin, suggesting the suppression of hepatic CSCs. Cell cycle regulation and apoptosis induction were synergistically enhanced when bestatin was combined with 5-fluorouracil, cisplatin, and doxorubicin. These effects were related to the increased intracellular oxidative stress caused by bestatin.

In the CD13+-enriched HCC tumor fraction obtained from 5-FU-treated mice serially implanted into secondary animals, tumor development was not found in bestatin-treated mice (*n* = 0/6), although 60% of untreated mice developed tumors (*n* = 6/10) [67]. In PDX models, bestatin combined with sorafenib inhibited tumor development and reduced HCC cell resistance to sorafenib, and no significant side effects were observed [68]. In an additional study, coadministration of bestatin with metronomic administration of low-dose cyclophosphamide caused decreased tumor growth and volume in an HCC xenograft model without significant toxicity [69].

Toshiyama et al. [70] developed a poly(ethylene glycol)-poly(lysine)-bestatin block copolymer conjugate to increase the efficacy of bestatin in suppressing HCC CSCs. This conjugate increased intracellular oxidative stress, leading to apoptosis in cultured HCC cells, thereby improving the antitumor effect in mice. Furthermore, Dou et al. [71] investigated the effects of BC-02, a conjugated compound of bestatin and 5-fluorouracil, on HCC CSCs. Compared with bestatin alone, BC-02 suppressed HCC CSCs more strongly, as did the combination of 5-fluorouracil and bestatin. The effects of BC-02 were supported by its ability to target CD13 and induce intracellular oxidative stress.

Zhang et al. [72] reported that the survival period of Kunming mice with HCC H22 cells after treatment with BC-02 was equivalent to that after treatment with capecitabine. Bestatin has also been reported to suppress leukemic stem cells from chronic myeloid leukemia [73] and mammary [74] CSCs.

#### Curcumin

Curcumin is a natural yellow polyphenolic pigment found in golden turmeric (*Curcuma longa*) and *Curcuma xanthorrhiza* oil, which are commonly used as spices and have low toxicity. Marquardt et al. [75] reported that curcumin-induced cell death in sensitive HCC cell lines was associated with NF-κB suppression and CSC depletion, whereas curcumin-resistant HCC cells, on the other hand, demonstrated unexpected increases in proliferation and CSC marker expression. When combined with a class I/II histone deacetylase (HDAC) inhibitor, trichostatin sensitized curcumin-resistant HCC cells, demonstrating that the CSC-depleting effect of curcumin involves NF-κB-mediated HDAC inhibition.

Wang et al. [76] demonstrated that curcumin suppressed HCC CSCs via inhibition of the PI3K/Akt/mTOR signaling pathway. Curcumin also suppresses or sensitizes CSCs from colorectal [77], prostate [78], lung [79], and mammary [80] cancers.

#### DAPT

DAPT is a Notch inhibitor that targets the γ-secretase complex. DAPT reduced EpCAM+ and EpCAM− Huh7 cell fractions and inhibited liver cancer growth, prolonging survival in an in vivo model [81]. DAPT also decreased the number of CD133+/EpCAM+ and CD90+ cells while downregulating the expression of stemness pathway genes (Wnt and Notch). In addition, DAPT therapy reduces the number and size of spheres generated by Huh7 HCC cells, as well as NANOG and SOX2 protein levels [82].

DAPT administration reduced Notch-related gene expression in HCC cells, resulting in a reduction in the CD133+ population induced by vincristine and 5-fluorouracil, triggering apoptosis, generating fewer spheroids in 3D culture and reducing migratory capacity [83]. CSCs from several other types of cancer, including ovarian [84], lymphoma [85], glioma [86], breast [87], lung [88], and gastric [89] cancer, have also been reported to be reduced or sensitized by DAPT.

#### IC-2

IC-2 is an inhibitor of the Wnt signaling pathway. It reduced the populations of human HCC cells that expressed the CSC markers CD44, CD90, CD133, and EpCAM, as well as their ability to form spheres. In vivo, IC-2 reduced tumor growth in mice xenografted with CD44-positive HuH7 cells [90]. Similarly, IC-2 inhibited and sensitized CSCs from colorectal [91] and oral squamous cell [92] cancers.

#### Isoxazole-piperazine analog

A series of isoxazole-piperazine analogs were prepared by İbiş et al. [93], in which 6a and 13d triggered G_1_ or G_2_/M arrest and induced apoptotic cell death in HCC cell lines. Importantly, these compounds reduced the fraction of CSCs (CD133+/EpCAM+), the expression of stemness markers (NANOG or OCT4 proteins), and the ability of HCC cells to form spheres.

#### LDN193189

LDN193189 is a small molecule with a potent inhibitory effect on the bone morphogenetic (BMP) pathway through the inhibition of activin receptor-like kinases 1, 2, 3 and 6. LDN193189 suppressed the interaction between CD133 and p85, and reduced the self-renewal and tumorigenicity of hepatic CSCs [94]. A drug delivery system consisting of Fe_3_O_4_ nanocubes carrying LDN193189 reversed EMT by targeting hepatic CSCs. Inhibition of the expression of the stemness-related genes OCT4 and NANOG and suppression of proliferation and migration via regulation of the expression of markers of the EMT process were observed [95].

LDN193189 also suppressed clonogenicity and decreased the CSC-enriched ALDH1+ population in malignant breast tumors, and in a mouse model of breast cancer treated with LDN193189, a reduction in the expression of EMT-associated markers was observed [96]. Similarly, LDN193189 suppressed CSCs from medulloblastoma [97] and endometrial [98] cancer.

#### Lupeol

Lupeol is a pentacyclic triterpene present in several edible fruits and vegetables. Lee et al. [99] reported that lupeol suppressed liver CSC spheroids derived from HCC cell lines and clinical specimens. Lupeol reduced the growth of the chemosensitive HCC cell lines Huh-7 and PLC-8024 in nude mice, suppressed the growth of MHCC-LM3 cells, a chemoresistant HCC cell line, in a xenograft model and decreased CD133 expression levels in tumors. Lupeol also increased the sensitivity of HCC cells to cisplatin and doxorubicin via the PTEN/Akt/ABCG2 pathway. Additionally, lupeol-induced inhibition of CSCs has also been demonstrated in prostate [100], retinoblastoma [101], melanoma [102] and colorectal [103] cancers.

#### Nitidine chloride

Nitidine chloride is a bioactive alkaloid isolated from the Chinese medicinal herb *Zanthoxylum nitidum* (Roxb.) DC. Importantly, nitidine chloride nanoparticles reduced HCC cell growth and suppressed the expression of aquaporin 3 and STAT3. It also decreased the fraction of EpCAM+/CD133+ HCC cells and inhibited sphere formation. Treatment with nitidine chloride nanoparticles suppressed Huh7 xenograft tumor development in HCC-bearing nude mice [104]. Nitidine chloride has also been reported to inhibit CSCs from colorectal [105], glioblastoma [106], and breast [107] cancers.

#### OSU-CG5

OSU-CG5 is an energy restriction mimetic agent that mimics glucose starvation and inhibits the mTOR pathway. OSU-CG5 decreased the CD90 population in primary liver tumor samples and liver cancer cell lines. It also inhibited tumor development in an animal model [108].

#### Phenyl-2-pyrimidinyl ketone 4-allyl-3-amino selenourea

Phenyl-2-pyrimidinyl ketone 4-allyl-3-amino selenourea was selected from a screening of 365 compounds that could inhibit HCC CSCs. This molecule binds to the c-MYC bHLH/LZ domains, preventing the development of the c-MYC-Max complex and its occupancy of target gene promoters. In animal models, it sensitized sorafenib-resistant tumors to sorafenib, decreased early tumor growth, suppressed CSC development, and showed antimetastatic activity [109].

#### Polyphyllins

Polyphyllins are bioactive compounds isolated from Rhizoma Paridis (Chonglou), a traditional Chinese medicine. Polyphyllin I inhibited HCC CSCs via Akt/GSK-3β/β-catenin signaling, as observed by ex vivo experiments, which revealed that polyphyllin I activated the Akt/GSK-3β-mediated ubiquitin-mediated proteasomal degradation of β-catenin and thereby inhibited the properties of HCC CSCs. It also reduced tumor growth in HCC cells in a xenograft model [110].

Xu et al. [111] demonstrated the effect of polyphyllin VII on HCC CSCs and explored its mechanism of action. The HCC cell lines Huh7 and HepG2 were used to investigate the antitumor activity of polyphyllin VII by quantifying cell growth and metastasis and to study its effects on stemness. Polyphyllin VII treatment induced apoptosis and inhibited proliferation, migration, tumor spheroid formation, and stemness (induced a reduction in the expression of the stemness markers OCT4 and CD133) in Huh7 and HepG2 cells. The anti-HCC CSC effects of polyphyllin VII are mediated by targeting the STAT3 signaling pathway. Zhu et al. [112] reported that polyphyllin VII can also suppress colorectal CSCs.

#### Resolvin D1

Resolvin D1 is an endogenous anti-inflammatory lipid mediator. Sun et al. [113] reported that the administration of resolvin D1 prevented cancer-associated fibroblast (CAF)-induced hepatic CSCs and the EMT of HCC cells in coculture and orthotopic liver tumor models. The action of resolvin D1 on CAFs is associated with reduced production of cartilage oligomeric matrix protein (COMP). Resolvin D1 affects CAF-derived COMP in a paracrine manner by inhibiting FPR2/ROS/FOXM1 signaling, eventually preventing FOXM1 recruitment to the COMP promoter.

#### RU-A1

RU-A1 is an inhibitor of B-cell-specific Moloney murine leukemia virus integration site 1 (BMI1). Bartucci et al. [114] demonstrated that RU-A1 reduces BMI1 expression, cell survival, and cell migration while sensitizing HCC cells to 5-fluorouracil. Notably, RU-A1 reduced the hepatic CSC population. Furthermore, zebrafish xenograft experiments demonstrated that RU-A1 inhibited tumor development in vivo.

#### Ruthenium complexes

Ruthenium-based complexes containing 2-thiouracil derivatives with the chemical formulas *trans*-[Ru(2TU)(PPh_3_)_2_(bipy)]PF_6_ and *trans*-[Ru(6m2TU)(PPh_3_)_2_(bipy)]PF_6_ (where 2TU = 2-thiouracil and 6m2TU = 6-methyl-2-thiouracil) inhibited HCC CSCs by targeting NF-κB and Akt/mTOR signaling, causing apoptotic cell death. A reduction in clonogenic potential, expression of CD133+ and CD44^high^ cells, development of tumor spheroids and cell motility were detected in HepG2 cells treated with these complexes. Tumor growth inhibition in HepG2 cell xenograft-bearing mice treated with these complexes was also observed, with a safe profile [115].

A ruthenium complex containing 1,3-thiazolidine-2-thione, with the chemical formula [Ru(tzdt)(bipy)(dppb)]PF_6_ (where tzdt = 1,3-thiazolidine-2-thione, bipy = 2,2’-bipyridine and dppb = 1,4-bis(diphenylphosphino)butane), suppressed hepatic CSCs in culture. This complex inhibited the clonogenic potential, CD133+ and CD44^high^ HepG2 cells, tumor spheroid growth and motility of HepG2 cells. The inhibition of Akt/mTOR signaling and the induction of apoptotic and autophagic cell death have also been reported [116]. Additionally, this molecule reduces the growth of HCC HepG2 cells in immunodeficient mice at a tolerable dose [117].

#### Salinomycin

Salinomycin is an ionophore antibacterial and coccidiostatic therapeutic drug and was originally identified as an anti-CSC drug in a screen of 16,000 chemicals, where it was selected as the only chemical that exhibited selectivity and potency in depleting mammary CSCs, which was related to its ability to increase the epithelial differentiation of tumor cells [118]. Sun et al. [119] reported that salinomycin suppressed the migration and invasion of hepatic CSCs by reducing the expression of the FAK-ERK1/2 signaling pathway, whereas Liu et al. [120] reported that salinomycin inhibited Wnt/β-catenin signaling, reducing the ability of hepatic CSCs both in vitro and in vivo.

Salinomycin also reduced hepatic CSCs from the HCC cell lines HepG2, SMMC-7721, and BEL-7402, resulting in apoptotic cell death. Salinomycin reduced the growth of liver tumors in an in vivo orthotopic HepG2 tumor model after 6 weeks of therapy, with doses of 4 and 8 mg/kg administered intraperitoneally [121]. Salinomycin combined with oncostatin M, a cytokine of the interleukin-6 family, reduced the CD133+ fractions of HepG2 cells, decreased cell invasion and stem cell-related gene expression, and triggered apoptosis in HCC HepG2 cells. This combination also modulates the production of AFP and albumin in HepG2 cells [122].

Nevertheless, the severe systemic toxicity of salinomycin limits its clinical use in humans. To overcome this, several nanoformulations have been developed to improve its safety and therapeutic efficacy. Internalizing Arg-Gly-Asp peptide-conjugated DSPE-PEG2000 nanomicelles for the delivery of salinomycin improved the cytotoxic impact on HCC CSCs, resulting in increased effectiveness of tumor penetration and anticancer activity in vivo [123]. Nanoliposomes containing salinomycin and doxorubicin suppressed tumor growth and reduced the proportion of hepatic CSCs in vivo [124], and liposomes containing salinomycin and chloroquine synergistically eradicated hepatic CSCs [125]. Salinomycin-loaded PEG-ceramide nanomicelles had greater cytotoxic effects and increased apoptosis-inducing activity in HCC CSCs, as well as an enhanced in vivo tumor suppressive effect and safety profile [126].

Nanocapsules of salinomycin combined with the modified linoleic acid prodrug 7-ethyl-10-hydroxycamptothecin increased apoptosis, inhibited tumor sphere formation, reduced cell motility and invasion, and decreased the proportion of HCC CSCs. In nude mice, these nanocapsules suppressed the growth of tumor xenografts derived from HCC cell lines and patients [127]. Furthermore, upconversion nanoparticles containing salinomycin and the photosensitive reagent iodide IR780 inhibited the migration of HCC CSCs via activation of the MAPK pathway [128].

Wang et al. [129] developed nanocarrier-based liposomes that selectively deliver doxorubicin and salinomycin to CD133+ EpCAM+ HCC CSCs. Interestingly, this system enhanced the elimination of both CSCs and non-CSCs from liver cancer cells both in vitro and in vivo. Salinomycin has also been shown to have anti-CSC effects on many other tumors, including breast [130], blood [131], lung [132], colorectal [133], osteosarcoma [134], and gastric [135] cancers.

#### SJB3-019A

Lu et al. [136] proposed a potential novel therapeutic strategy to target hepatic CSCs via ubiquitin-specific protease 1 (USP1), which plays a key role in the development of different cancers. The USP1 inhibitor SJB3-019A sensitized stem-like and nonstem-like HCC cells to doxorubicin by increasing the ubiquitylation of proliferating cell nuclear antigen. Doxorubicin/SJB3-019A increased apoptosis in primary liver carcinoma and MHCC-97H cell line and induced tumor inhibition in in vivo liver tumor models. Furthermore, doxorubicin/SJB3-019A treatment decreased the sphere-forming ability and the expression of NANOG, SOX2, and c-MYC, reducing cancer stemness. Interestingly, Das et al. [137] reported that SJB3-019A can also suppress the CSCs of multiple myeloma cells.

### Small molecules in the clinical development stage

Three small molecules targeting HCC CSCs (arsenic trioxide, galunisertib, and metformin) have been evaluated in clinical trials registered on the ClinicalTrials.gov website and are highlighted in Table 3. Additionally, many authors have reported clinical trials, pilot studies, or case reports on the clinical use of all-trans retinoic acid, olaparib, and RO4929097 in humans. These molecules are discussed in this section.

**Table 3:** Small molecules that target HCC CSCs and are being evaluated in clinical trials as anti-HCC agents^a^.

ClinicalTrials.gov ID	Official title	Conditions	Intervention/treatment	Phase	Study start	Current status
NCT02956772	Transcatheter arterial chemoembolization (TACE) in combination with arsenic trioxide versus tace in the treatment of middle-advanced primary hepatocellular carcinoma (HCC) patients	Primary hepatocellular carcinoma	Drug: TACE Drug: Arsenic trioxide	Phase 2	2016-11	Unknown status
NCT00582400	A phase II protocol of arsenic trioxide (Trisenox) in subjects with advanced primary carcinoma of the liver	Carcinoma, Hepatocellular	Drug: arsenic trioxide	Phase 2	2004-09	Terminated -recruiting or enrolling participants has halted prematurely and will not resume; participants are no longer being examined or treated
NCT02018757	Clinical application study of transarterial chemoembolization containing arsenic trioxide in the treatment of hepatocellular carcinoma	Carcinoma, Hepatocellular	Drug: TACE containing As2O3 Drug: TACE containing placebo	Phase 2	2014-01	Unknown status
NCT01861912	Arsenic trioxide TACE and intravenous administration compared with arsenic trioxide TACE alone in unresectable hepatocellular carcinoma: a randomized, parallel, controlled, multicenter clinical study	Carcinoma, Hepatocellular	Device: Arsenic trioxide TACE Drug: Arsenic trioxide intravenous infusion Drug: lipiodol Drug: NaCl solution	Phase 2 Phase 3	2013-06	Unknown status
NCT00128596	A phase II study of trisenox (arsenic trioxide) in the treatment of unresectable liver cancer	Liver Cancer	Drug: arsenic trioxide	Phase 2	2004-06	Completed
NCT02906397	A pilot study of galunisertib (LY2157299) plus stereotactic body radiotherapy (SBRT) in patients with advanced hepatocellular carcinoma (HCC)	Advanced hepatocellular carcinoma (HCC)	Drug: Galunisertib 150 mg by mouth twice a day Radiation: Stereotactic Body Radiotherapy (SBRT)	Phase 1	2017-03-30	Completed
NCT02240433	A phase 1b study of LY2157299 in combination with sorafenib in patients with unresectable hepatocellular carcinoma	Hepatocellular Carcinoma	Drug: LY2157299 Drug: Sorafenib	Phase 1	2014-11-12	Completed
NCT01246986	Phase 2 study of LY2157299 in patients with hepatocellular carcinoma	Carcinoma, Hepatocellular	Drug: LY2157299 Drug: Sorafenib Drug: Ramucirumab	Phase 2	2011-03-30	Completed
NCT02178358	A randomized phase 2 study of LY2157299 versus LY2157299 - sorafenib combination versus sorafenib in patients with advanced hepatocellular carcinoma	Hepatocellular Carcinoma	Drug: LY2157299 Drug: Sorafenib Drug: Placebo	Phase 2	2014-08-08	Completed
NCT02423343	A phase 1b/2 dose escalation and cohort expansion study of the safety, tolerability and efficacy of a novel transforming growth factor-beta receptor I kinase inhibitor (galunisertib) administered in combination with anti-PD-1 (nivolumab) in advanced refractory solid tumors (phase 1b) and in recurrent or refractory non-small cell lung cancer or hepatocellular carcinoma (phase 2)	Solid Tumor Non-Small Cell Lung Cancer Recurrent Hepatocellular Carcinoma Recurrent	Drug: Galunisertib Drug: Nivolumab	Phase 1 Phase 2	2015-01-01	Completed
NCT02672488	Safety and efficacy of metformin plus sorafenib as first-line therapy in patients with advanced hepatocellular carcinoma (BCLC-C): a phase 2 randomized study	Hepatocellular Carcinoma	Drug: Sorafenib Drug: Metformin	Phase 2	2015-12	Unknown status
NCT02819869	The combination effect of statin plus metformin on relapse-free	Hepatocellular Carcinoma	Drug: Statin and Metformin use	Phase 2	2016-08-01	Terminated -insufficient for the fund
NCT02319200	Primary prevention of hepatocellular carcinoma by metformin in patients with viral c cirrhosis: prospective multicenter study, randomized control trial. Ancillary Study of the ANRS CO12 CirVir Cohort	Hepatocellular Carcinoma Hepatitis C, Chronic Cirrhosis	Drug: Metformin Drug: placebo tablet	Phase 3	2015-06	Terminated Decision of investigator
NCT03184493	Celebrex and metformin for postoperative hepatocellular carcinoma	Liver Cancer	Drug: Celebrex plus Metformin Drug: Celebrex Drug: Metformin	Phase 3	2017-06-02	Unknown status
NCT04033107	Clinical research of high dose vitamin C combined with metformin in the treatment of malignant tumors	Hepatocellular Cancer Pancreatic Cancer Gastric Cancer Colorectal Cancer	Drug: Vitamin C Drug: Metformin	Phase 2	2020-07-01	Recruiting
NCT04114136	A phase ii clinical trial of anti-PD-1 mAb therapy alone or with metabolic modulators to reverse tumor hypoxia and immune dysfunction in solid tumor malignancies	Melanoma NSCLC Hepatocellular Carcinoma Urothelial Cancer Gastric Adenocarcinoma HNSCC Esophageal Adenocarcinoma Microsatellite Instability-High Solid Malignant Tumor	Drug: Nivolumab or Pembrolizumab (dependent upon approved indication) Drug: Metformin Drug: Rosiglitazone	Phase 2	2020-09-14	Recruiting

^a^These data were obtained from www.clinicaltrials.gov on June 17, 2025, via the search term “HCC” and the drugs selected in Table 2.

#### All-trans retinoic acid

All-trans retinoic acid, also known as tretinoin, is a retinoid derivative (vitamin A analog) that is a clinically approved drug for treating acne and acute promyelocytic leukemia. Zhang et al. [138] reported that all-trans retinoic acid induced hepatic CSC differentiation and potentiated the cytotoxic effects of cisplatin by suppressing Akt phosphorylation. The combination therapy inhibited HCC cell migration in vitro and metastasis in vivo more strongly than either therapy alone. Zhu et al. [139] demonstrated that all-trans retinoic acid suppressed PI3K/Akt activity and increased the GSK-3β-dependent degradation of phosphorylated β-catenin, resulting in the differentiation of hepatic CSCs and increasing their sensitivity to docetaxel.

All-trans retinoic acid treatment reduced sorafenib-induced EpCAM+ cells and clonal growth via the inhibition of Akt phosphorylation in Huh7 HCC cells, thereby eradicating sorafenib resistance in culture and in the PDX model [140]. Similarly, all-trans retinoic acid treatment reduces the stem cell formation capacity and vasculogenic mimicry of HCC cells in culture and animal models [141]. The combination of all-trans retinoic acid and doxorubicin inhibited HCC metastasis in the LM3/miR-452 mouse model and suppressed the percentage of CD133+ cells [142].

CSCs from lung [143], cervical [144], glioblastoma [145], thyroid [146], head and neck [147], ovarian [148], breast [149], colorectal [150] and gastric [151] cancers have also been shown to be sensitive to all-trans retinoic acid.

In a phase 2 clinical trial, 29 chemotherapy-naïve patients with primary HCC received oral all-trans retinoic acid monotherapy. All-trans retinoic acid (50 mg/m^2^ t.i.d.) was administered 3 weeks on/1 week off cycle until progression or grade 3 or 4 toxicity. However, this treatment was ineffective and included a wide range of toxicities, such as headache, fatigue, nausea, vomiting, mucositis, and neurotoxicity [152]. On the other hand, a pilot clinical trial conducted in 15 patients with advanced HCC evaluated the efficacy of all-trans retinoic acid combined with tamoxifen and vitamin E and reported that this combination increased survival rates and improved the clinical outcomes of these patients. No patients reported severe side effects enough to require withdrawal from the study. Side effects included headache, mucositis, pain, and asthenia [153].

A retrospective study compared 5-fluorouracil/leucovorin/oxaliplatin (FOLFOX4) to all-trans retinoic acid with FOLFOX4 treatment in individuals with advanced HCC. This study included 111 patients, and the all-trans retinoic acid plus FOLFOX4 group had a longer median time to progression than the FOLFOX4 group did (3.6 months vs. 1.8 months) [154]. Another retrospective analysis comparing FOLFOX4 or all-trans retinoic acid plus FOLFOX4 in 66 patients with advanced HCC revealed that the all-trans retinoic acid plus FOLFOX4 group had a longer median survival (14.0 vs 8.0 months) and median time to progression (8.7 vs 3.2 months) [155].

A randomized, placebo-controlled, double-blind, multicenter clinical trial enrolled a total of 108 patients to receive FOLFOX4 plus all-trans retinoic acid or FOLFOX4 placebo. The FOLFOX4 plus all-trans retinoic acid group had a median progression-free survival of 7.1 months, whereas the FOLFOX4-placebo group had a median progression-free survival of 4.2 months, indicating that FOLFOX4 combined with all-trans retinoic acid is safe and effective for individuals with advanced HCC [156].

Overall, these preclinical and clinical data highlight the anti-HCC potential of all-trans retinoic acid, and further clinical trials, including phase 3 clinical trial, should be considered to evaluate the optimal therapeutic regimen for this drug in HCC patients.

#### Arsenic trioxide

Arsenic trioxide, an inorganic compound with the formula As_2_O_3_, is an ancient medicine that has been investigated as a therapeutic agent for the treatment of different types of cancer, although it is classified as carcinogenic to humans. Wang et al. [157] investigated arsenic trioxide against liver CSCs and reported that the induction of DNA demethylation after arsenic trioxide treatment activated microRNA-148a, leading to the inhibition of the NF-κB pathway and CSC properties in the multidrug-resistant HCC cell line MDR Bel-7402 and the resensitization of these cells to the drug 5-fluorouracil. Li et al. [158] reported that arsenic trioxide elevated miR-491 expression via DNA demethylation, causing reduced SMAD3 expression and suppressing CSC-like features in HCC MHCC97H cells in vitro and in a mouse xenograft model.

Furthermore, arsenic trioxide caused HCC CSC differentiation by targeting GLI1 expression in a mouse model [159]. Similarly, Zhang et al. [160] demonstrated that arsenic trioxide caused hepatic CSC differentiation by inhibiting the LIF/JAK1/STAT3 and NF-κB signaling pathways, whereas Wang et al. [161] reported that arsenic trioxide suppressed hepatic CSCs and metastasis by targeting the SRF/MCM7 complex.

In the HCC cell lines MHCC97L and Hep3b, arsenic trioxide-based nanoparticles reduce tumor spheroid formation in vitro and tumor initiation in vivo by inhibiting the expression of hepatic CSC markers (CD133, SOX2, and OCT4), modulating EMT indicators (E-cadherin, vimentin, and Slug), and regulating the SHP-1/JAK2/STAT3 signaling pathway [162]. Interestingly, arsenic trioxide also affects CSCs from glioma [163], pancreas [164], nasopharyngeal [165], and lung [166] cancers.

A phase 2 clinical trial conducted in 29 patients with advanced HCC revealed no advantage when a total of 61 cycles of arsenic trioxide were administered at dosages of 0.16–0.24 mg/kg per day for 5–6 days per week for 3–4 weeks, followed by a week of rest. The toxicity associated with this dosing schedule is predominantly hematologic, including leukopenia and neutropenia, which are manageable and do not lead to hospitalization [167]. On the other hand, arsenic trioxide has shown promising results in the treatment of HCC, with an analgesic effect, in a multicenter phase 2 clinical trial where arsenic trioxide was injected into 102 patients with HCC at a dosage of 7–8 mg/m^2^ i.v. qd for 14 days (repeated after 7–14 days) and evaluated after two treatment cycles. The main side effects are gastrointestinal reactions and bone marrow suppression [168].

A randomized controlled clinical trial investigated the efficacy of arsenic trioxide combined with transcatheter arterial chemoembolization (TACE) in the treatment of primary liver cancer with lung metastases. A total of 30 patients received TACE with 10 mg arsenic trioxide by intravenous infusion for 5 h daily, 3 days after TACE (each cycle consisted of 14 days of administration and was repeated after 2 weeks, with each patient receiving 3–4 cycles). The control group included 30 subjects who received TACE alone. The quality of life improved in four patients and remained stable in 18 patients in the arsenic trioxide group, whereas no patients improved, and 13 patients remained stable in the control group [169].

A randomized controlled clinical trial of TACE combined with arsenic trioxide was conducted in 125 patients with HCC. All patients received TACE, and 61 patients received arsenic trioxide at 10 mg/d for 4 cycles (21 days per cycle) with a 2-week interval between cycles. TACE combined with arsenic trioxide treatment prevented extrahepatic metastasis and prolonged survival in patients with HCC. Regarding side effects, no significant differences were found between the groups in hematologic or digestive system disorders or liver or kidney dysfunction, but treatment with arsenic trioxide increased the risk of facial edema and skin rash [170].

Similarly, a randomized clinical trial examined the use of intravenous arsenic trioxide in combination with TACE to treat patients with advanced HCC with lung metastases. Patients treated with TACE plus arsenic trioxide were compared with those treated with TACE alone. The 1- and 2-year overall survival rates in the TACE plus arsenic trioxide group were 56.7% and 16.7%, respectively, whereas those in the TACE alone group were 36.7% and 3.3%, demonstrating that patients with advanced HCC who received TACE plus arsenic trioxide had longer overall survival than those who received TACE alone. Treatment including arsenic trioxide appeared to be associated with an increased risk of adverse events such as facial edema, gastrointestinal reactions, and leukopenia [171].

A randomized, single-blind clinical trial was conducted in patients with unresectable HCC and lung metastases, in which TACE with arsenic trioxide (*n* = 69) was compared with TACE with arsenic trioxide plus additional intravenous arsenic trioxide administration (*n* = 70). The median overall survival was 7.3 months in the group that received TACE with arsenic trioxide plus intravenous arsenic trioxide, whereas it was 2.9 months in the control group. Adverse effects associated with arsenic trioxide therapy were considered to be of low intensity and included myocardial ischemia, fever, thrombocytopenia, leukopenia, rash, nausea, vomiting, bloating, diarrhea, and headache [172]. Moreover, Lv et al. [173] reported that adjuvant arsenic trioxide therapy combined with TACE has superior therapeutic outcomes to TACE alone in a meta-analysis of 1412 patients with primary liver cancer.

A randomized controlled clinical trial enrolled a total of 207 patients to compare the effects of TACE with CalliSpheres® arsenic trioxide-loaded spheres (CBATO-TACE) to those of TACE with arsenic trioxide/lipiodol emulsion (standard TACE) in the first-line treatment of patients with large HCC (5 cm ≤ maximum diameter < 10 cm) or enormous HCC (maximum diameter ≥ 10 cm). The median progression-free survival in the CBATO-TACE group was 9.5 months, whereas it was 6.0 months in the standard TACE group. Patients in the CBATO-TACE group had a median overall survival of 22 months, whereas those in the standard TACE group had a median overall survival of 16 months, indicating a better outcome in the CBATO-TACE group. In addition, CBATO-TACE treatment was well tolerated at a similar or better level than standard TACE [174].

Similarly, a controlled clinical investigation evaluated the effects of CBATO-TACE (*n* = 38) on arsenic trioxide/lipiodol emulsions (standard TACE) (*n* = 48) in patients with unresectable HCC. The CBATO-TACE group had a median progression-free survival and overall survival of 308 and 548 days, respectively, whereas the standard TACE group had durations of 148 and 404 days, respectively. Interestingly, the CBATO-TACE group showed equal tolerance to the standard TACE group [175].

Arsenic trioxide has also demonstrated some beneficial clinical effects when combined with standard chemotherapies in patients with acute promyelocytic leukemia [176], neuroblastoma [177], lymphoma [178], and multiple myeloma [179].

These data identify arsenic trioxide as an important chemotherapeutic agent against multiple cancers. The anti-HCC effect of arsenic trioxide should be explored further to improve the survival and quality of life of patients.

#### Galunisertib

Galunisertib, also known as LY2157299, is a selective ATP-mimetic inhibitor of the TGF-β receptor I. Galunisertib decreased the expression of stemness-related genes in both HCC cells and ex vivo human HCC tissues. Furthermore, galunisertib inhibited stemness-related activities in invasive HCC cells, reducing colony formation, hepatic spheroid formation, and invasive growth ability [180]. Galunisertib effectively suppressed CK19+ HCC cells in vitro and in vivo [181]. Galunisertib also affects neuroblastoma [182], breast cancer [183], and pancreatic [184] CSCs.

An open-label, multicenter, phase 2 clinical trial of galunisertib combined with sorafenib (400 mg) conducted in patients with advanced HCC reported satisfactory safety and prolonged overall survival. Galunisertib was tested at 80 or 150 mg orally for 14 days every 28 days. The most common side effects were palmar-plantar erythrodysesthesia, diarrhea, and pruritus [185]. In addition, a phase 1b open-label clinical trial of galunisertib plus ramucirumab, an anti-VEGF receptor 2 antibody, in patients with advanced HCC revealed that the maximum tolerable dose of galunisertib was 150 mg orally twice daily and 8 mg/kg intravenously every other week [186]. A phase 1b clinical trial in Japanese patients with unresectable HCC revealed a safety profile for galunisertib (150 mg twice daily for 14 days) plus sorafenib [187].

Galunisertib was evaluated in an open-label phase 2 clinical trial in patients with HCC and had a reasonable safety profile, while lower AFP and TGF-β1 levels were associated with longer survival. The most frequent adverse events of moderate/high intensity were neutropenia, fatigue, anemia, increased bilirubin, hypoalbuminemia and embolism [188]. Likewise, another phase 2 clinical trial demonstrated that circulating AFP and TGF-β1 levels predict survival in patients with advanced HCC treated with galunisertib [189]. Furthermore, some clinical trials have reported important benefits for patients with lung [190], pancreas [191] and rectal [192] cancers who received galunisertib along with standard chemotherapies.

Therefore, the anti-HCC effect of galunisertib should be further investigated in preclinical and clinical studies to determine the best therapeutic regimen with the ability to improve the survival and quality of life of patients with HCC.

#### Metformin

Metformin, an antidiabetic drug with a safe profile, has cytotoxic effects on several types of cancer. It acts as an inhibitor of oxidative phosphorylation. Liver CSCs isolated from HCC biopsies were treated with metformin, a cisplatin/interferon α-2b/doxorubicin/5-fluorouracil (PIAF) chemotherapy regimen, or a combination of these two protocols. Metformin alone and in combination with PIAF decreased the proliferation of liver CSCs and increased oxidative stress by decreasing the reduced glutathione levels and increasing the lipid peroxide levels. Furthermore, increased apoptosis via oxidative stress and the induction of autophagy in tumor cells treated with metformin and the PIAF regimen have also been reported [193].

Metformin reduced CD133 expression in the HCC cell lines HepG2, JHH6, JHH7, and Huh1, and these effects were associated with the adenosine monophosphate (AMP)-activated protein kinase (AMPK)/CCAAT/enhancer-binding protein beta (CEBPβ) signaling pathway. Metformin treatment also inhibited the phosphorylation of p70-S6 kinase, a typical mTOR substrate, as well as the phosphorylation of STAT3 in HCC cells. Additionally, metformin sensitized resistant HCC cells to the chemotherapy drug 5-fluorouracil [194].

Metformin also suppressed the in vivo growth of residual heat-exposed HCC cells by inhibiting POSTN secretion and reducing the expression of CSC markers [195]. The combination of metformin and doxorubicin led to doxorubicin sensitization in R-HepG2 cells, a resistant HCC cell line with a CSC phenotype [196]. On the other hand, Xin et al. [197] reported that metformin enhanced the effects of sorafenib on HCC while increasing the CSC population.

Metformin also inhibited spheroids and the expression of markers related to the CSC and EMT phenotype in cholangiocarcinoma cells in cell culture and reduced tumor growth in animals bearing tumor xenografts [198].

The ability of metformin to selectively eliminate mammary CSCs has also been reported [199, 200]. Similar results have also been reported in leukemia [201], colorectal [202], gastric [203], osteosarcoma [204], glioblastoma [205], ovarian [206], pancreas [207], lung [208], endometrial [209], esophageal [210], and oral [211] cancer cells.

In a meta-analysis by Kramer et al. [212], metformin use was associated with a 20% lower risk of developing HCC in patients with diabetes. Furthermore, several other meta-analyses have shown that metformin reduces the risk of developing HCC [213, 214] or improves the survival of patients with HCC [215–217]. In contrast, a retrospective clinical investigation of 857 HCC patients who underwent primary resection and used metformin for diabetes mellitus revealed that metformin had no protective effect on HCC recurrence [218].

The effect of metformin in patients with HCC was also evaluated in a retrospective cohort study by a Korean multicenter including 1566 patients with unresectable HCC who received sorafenib treatment. Patients who receive metformin have improved long-term survival, suggesting advantages for HCC patients treated with metformin in combination with sorafenib [219]. Similar results were observed in the USA [220], Taiwan [221], and the UK [222] but not in Malaysia, where metformin was associated with a poor prognosis for HCC survival [223]. In contrast, a prospective, randomized clinical trial of 80 patients with advanced HCC treated with sorafenib plus metformin or sorafenib alone revealed that adding metformin to sorafenib did not improve treatment efficacy in patients with HCC [224].

Furthermore, a phase 1 clinical trial evaluating a dose reduction of sorafenib (800 mg, 600 mg, 400 mg, and 200 mg) in combination with atorvastatin (10 mg) and metformin (500 mg) in patients with advanced HCC reported a decrease in sorafenib-related side effects. The authors hypothesized that this reduction in sorafenib-related side effects may be due to balanced sorafenib exposure and increased tolerance, which are mediated by drug interactions with metformin and/or atorvastatin [225].

Metformin has also been shown to improve therapy for patients with ovarian [226], lymphoma [227], breast [228], prostate [229], and lung [230] cancers.

These findings suggest that metformin could be used in adjuvant cancer therapy, including for individuals with HCC. Additional preclinical and clinical studies should confirm the antineoplastic activity of metformin.

#### Olaparib

Olaparib is a poly(ADP-ribose) polymerase (PARP) inhibitor approved to treat BRCA1/2-mutated tumors, including ovarian, breast, pancreatic, and prostate cancers. Surprisingly, PARP1 overexpression was detected in HCC patients and was associated with poor clinical prognosis. Moreover, in a sorafenib-treated xenograft model, the remaining HCC tumors reactivated PARP1 expression. Notably, olaparib treatment suppressed pluripotent transcription factor (SOX2, OCT4, and c-MYC) and DNA damage repair signaling via CHD1L-mediated structural chromatin condensation at their promoters. Olaparib reduces tumorigenesis in HCC and enhances the effect of sorafenib both in vitro and in vivo [231].

Olaparib also kills or sensitizes CSCs from breast [232], glioblastoma [233], and colorectal [234] tissues. Conversely, olaparib enriched the ovarian CSC population after treatment [235].

Zhao et al. [236] reported a patient with liver cancer harboring a BRCA2 germline mutation who was treated with olaparib combined with nivolumab, an anti-programmed death-1 (PD-1) monoclonal antibody. The patient had stable disease and improved liver disease within 3 months of treatment. Su et al. [237] reported the case of a patient with metastatic combined hepatocellular cholangiocarcinoma harboring four clinically relevant single nucleotide variants (including BRCA2), two amplified genomic regions, and 11 heterozygous genomic deletions (including BRCA2) who was treated with olaparib and had a good outcome.

Li et al. [238] reported a patient with a secondary ovarian tumor with metastasis from primary liver cancer to the ovaries and omentum, harboring a BRCA2 mutation. After surgery and olaparib therapy, the blood levels of cancer antigen 125 (CA125) and AFP decreased and remained mostly within normal limits. However, she developed asthenia and severe leukopenia after olaparib administration, and treatment was discontinued after 2.5 months of use because the patient could not tolerate these side effects. On the other hand, Joris et al. [239] conducted a Belgian precision tumor-agnostic phase 2 study including a patient with advanced liver carcinoma with a BRCA1 mutation. However, treatment with olaparib did not halt disease progression.

Further preclinical and clinical studies should be conducted with olaparib to better understand its potential against HCC and other tumors, including its effects on CSCs. These studies should explore olaparib as monotherapy and in combination with agents such as bevacizumab, which is already approved for the treatment of other types of cancer.

#### RO4929097

RO4929097, also known as RG-4733, is a Notch inhibitor that targets γ-secretase. Jung et al. [240] demonstrated that RO4929097 (10 mg/kg) treatment in *Pten* null mice decreased/prevented liver tumor growth and liver fibrosis and reduced the expression of the hepatic progenitor cell markers CD24 and osteopontin, as well as the biliary cell markers CK19 and SOX9. RO4929097 treatment also resulted in the accumulation of A6+/EpCAM− cells, indicating that progenitor cells and newly generated hepatocytes lack CSC markers.

In addition, RO4929097 has been reported to reduce the number of CSCs from melanoma [241] and multiple myeloma [242]. In contrast, RO4929097 promoted CSC proliferation in breast cancer [243].

In a phase 1b clinical trial of RO4929097 combined with temsirolimus, an inhibitor of mTOR, in patients with advanced solid tumors, including patients with advanced HCC, RO4929097 was considered safe when combined with temsirolimus. The recommended phase 2 dose is 20 mg of RO4929097 combined with 37.5 mg of temsirolimus. The most common toxicities related to this combination were fatigue, mucositis, neutropenia, anemia, and hypertriglyceridemia [244].

The clinical development of RO4929097 was halted because of its undesirable pharmacological profile [244]; however, new Notch-targeted drugs should be studied for the treatment of HCC because of the critical role of the Notch signaling pathway in hepatic CSCs.

## Future perspectives and conclusions

This review presents a comprehensive overview of emerging small molecules that target HCC CSCs. Although a large body of evidence indicates that liver CSCs are important targets for improving the treatment of patients with liver cancer, only a few compounds have been evaluated in preclinical and especially clinical trials. Some important gaps in the development of anti-CSC drugs should be reconsidered, since most of the compounds tested in preclinical models have not been subjected to clinical trials.

Among a total of 30 small molecules with evidence of action on HCC CSCs, only six (all-trans retinoic acid, arsenic trioxide, galunisertib, metformin, olaparib and RO4929097) have been evaluated in clinical trials, pilot studies or case reports. Among them, all-trans retinoic acid combined with tamoxifen and vitamin E [153] or FOLFOX4 [154–156] has shown better clinical outcomes in patients with advanced HCC, highlighting that additional clinical studies, including phase 3 clinical trials, should be conducted to determine the appropriate therapeutic regimen for all-trans retinoic acid in patients with HCC.

Arsenic trioxide combined with TACE prevents extrahepatic metastases and prolongs survival time or improves quality of life in patients with HCC [169–173], and arsenic trioxide injection has shown promising results in the treatment of HCC, with an analgesic effect [168]. Furthermore, TACE with arsenic trioxide plus additional intravenous arsenic trioxide administration has a greater overall survival rate than TACE with arsenic trioxide does [172], whereas CBATO-TACE has better outcomes than TACE with arsenic trioxide/lipiodol emulsions does [174, 175]. These findings indicate the importance of arsenic trioxide as an anti-HCC treatment, and future clinical trials should be conducted to optimize its action in HCC with the aim of increasing survival and improving the quality of life of patients.

Treatment combining galunisertib and sorafenib or ramucirumab has demonstrated acceptable safety [186, 187] and prolonged the overall survival of patients with advanced HCC [185], where blood levels of AFP and TGF-β1 predict survival in patients with advanced HCC treated with galunisertib [188, 189]. These findings encourage further clinical trials with galunisertib in patients with HCC to identify the most effective regimen capable of improving survival and quality of life.

Additionally, the use of metformin in patients with HCC has been linked to increased long-term survival and a reduction in sorafenib-related side effects [219–222, 225]. Although some studies have not reported this association [218, 223, 224], the large number of positive results support further preclinical and clinical trials of metformin in patients with HCC to improve their quality of life and increase their survival.

Three case studies have reported the benefits of olaparib for patients with liver cancer [236–238]; however, a Belgian phase 2 study conducted in one patient with advanced BRCA1-mutated liver carcinoma concluded that olaparib treatment did not affect disease progression [239]. As a result, additional preclinical and clinical studies using olaparib are needed to better characterize its influence on CSCs as well as its benefit for HCC patients.

The Notch-targeted drug RO4929097 is considered safe in combination with temsirolimus in patients with advanced HCC; however, owing to its poor pharmacological profile, RO4929097 is no longer in clinical development [244]. In any case, new Notch-targeted drugs should be evaluated in preclinical and clinical trials in HCC patients to better understand their potential benefits.

Like RO4929097, several other compounds that target important cellular signaling pathways—such as Notch, Wnt/β-catenin, JAK/STAT, NF-κB, Hedgehog, PI3K/Akt/mTOR, and TGF/SMAD—have been shown to target CSCs in various cancer types [48–50]. A critical consideration in the development of signaling pathway inhibitors is the complex interconnectivity between these pathways within tumors, including CSCs. Selective inhibition of a single pathway can often trigger compensatory activation of alternative signaling pathways, ultimately decreasing therapeutic efficacy [245–250]. In these settings, multitarget strategies—whether through dual-specificity drugs or rational drug combinations—are often more effective and better suited to overcome pathway redundancy and resistance mechanisms.

Overall, anti-CSC drugs appear to have high potential for improving both quality of life and survival in the treatment of HCC patients in the future; however, more preclinical and clinical research is needed to better understand their effects and select the optimal therapeutic regimen. Furthermore, given the high complexity of CSC regulation, strategies that target CSCs should also include multiple targets, considering both the intracellular signaling pathways that regulate CSCs and extracellular factors. These therapies must also consider the ability of non-CSCs to dedifferentiate into CSCs under certain conditions, thus repopulating this target population. Thus, the most successful therapy should eliminate both CSCs and non-CSCs.

The development of these drugs should also consider potential off-target effects, drug resistance mechanisms or the plasticity of CSC populations, as well as combination therapies, the development of biomarkers for patient stratification or the potential of new drug delivery systems to increase the efficacy of these small molecules. This review emphasizes the need for further preclinical and clinical research to identify innovative strategies for combating liver cancer. By focusing on the potential to eradicate CSCs, we can increase treatment efficacy and ultimately improve the quality of life for patients facing this disease.
